# Macrostomia: A Review of Evolution of Surgical Techniques

**DOI:** 10.1155/2014/471353

**Published:** 2014-09-29

**Authors:** Srikanth Gunturu, Ranganadh Nallamothu, Rama Mohan Kodali, Koteswara Rao Nadella, Leela Krishna Guttikonda, Vijayalakshmi Uppaluru

**Affiliations:** Department of Oral and Maxillofacial Surgery, Drs Sudha and Nageswara Rao Siddhartha Institute of Dental Sciences, Chinnaoutpalli, Gannavaram, Andhra Pradesh 521286, India

## Abstract

Macrostomia is a congenital deformity resulting from failure of fusion of maxillary and mandibular process. It is a rare congenital deformity with an incidence of 1 in 60,000 to 1 in 300,000 live births. Transverse facial clefts are more common on right side of face in unilateral cases. Males are more affected than females. Various surgical techniques have been described in the literature for the correction of these defects. We report a case of macrostomia corrected with Z-plasty closure for skin, overlapping muscle closure, and triangular mucosal flap for commissure, with a review on existing techniques.

## 1. Introduction

Macrostomia is a rare congenital deformity with an incidence of 1 in 60,000 to 1 in 300,000 live births [1, 2]. Transverse facial clefts are more common on right side of face in unilateral cases [3]. Males are more affected than females. It results from failure of fusion of maxillary and mandibular process [4]. Gorlin believes that the lateral facial clefts are postmerging tears as there is considerable clinical variation [3]. Hartsfield and Bixler in their case report in one of monozygotic twins explained the role of multifactorial inheritance for the etiology [5]. Tessier's classification of facial clefts lists the macrostomia as number 7 [6]. It may be unilateral or bilateral, partial or complete extending up to tragus, and isolated or associated with syndromes. Treacher-collins syndrome and hemifacial microsomia [7] are frequently associated with macrostomia [8]. Problems associated with macrostomia include aesthetic disharmony and functional problems like feeding difficulties, drooling, speech incoherence, and difficulty in blowing. The goal of surgical correction of these clefts includes good aesthetics and better function of orbicularis oris muscle. The aesthetic outcome of these surgeries depends not only on the placement of scars along the natural skin creases but also on their showup during facial expressions.

Various surgical techniques have been evolved over a period of time with revisions to the existing ones to attain harmony between function and aesthetics. Surgical technique for the correction of macrostomia should address skin, muscle, and mucosa. There should be natural blending of the mucosa with the skin at the commissure. For commissure, triangular mucosal flaps or triangular skin flaps are used. For skin closure, straight line or Z- or W-plasty [9, 10] is used. Straight line muscle closure or overlapping myoplasties are used for muscle reconstruction. Complications observed with surgical techniques include asymmetric closure, hypertrophic scar, drooping of oral commissure, and fish mouth deformity resulting from flaccid commissure. One should consider the symmetry both in vertical and in horizontal plane as improper techniques might result in asymmetry [8].

Yoshimura et al. [11] in 1992 suggested positioning the commissure as described by Boo-Chai in 1969 [12] which is based on careful observation of the change of texture of the vermilion from normal skin to cleft mucosa and the use of measurements for symmetry without considering the change of texture results in poor aesthetics. Straight line closure of orbicularis oris muscle gives pursed lips; to avoid this, Kaplan [13, 14] in 1981 suggested overlapping myoplasty for muscle closure. Yoshimura et al. [11] in 1992 suggested small triangular skin flaps [15] from lower lip to be transposed into the commissure, to resemble natural overlap of upper lip over the lower lip as described by Onizuka in 1965 [16]. Kajikawa et al. suggested oblique vermilion mucosal incisions for the commissural reconstruction [2]. Z-plasties were suggested for skin closure to make the scars inconspicuous and to avoid contracture. But some authors claim that Z-plasty causes downward and lateral migration of reconstructed commissure; to this, Mc Carthy [17] suggested incorporating the Z-plasty in which the central limb falls on the nasolabial fold [18]. Kawai et al. [17] suggested the straight line closure with simple excision of the dog ear for the skin. Eguchi [19, 20] repaired the macrostomia using vermilion square flap technique which combines a lower lip mucocutaneous vermilion border flap with a lazy W-plasty to ensure natural commissure and cheek skin closure. Straight line closure results in formation of dog ear as the lengths of upper and lower incisions were uneven [11]. Z-plasty aids in lengthening the transverse deficient cheek. Vermilion square flap [19, 20] prevents migration of commissure laterally and it prevents linear contracture of scar as well. Both Z-plasty and vermilion square flap methods are technique sensitive and require meticulous execution in order to avoid unsightly scar. Straight line closures are ideal where transverse deficiency of cheek is minimal.

## 2. Case Report

**Figure 1:**
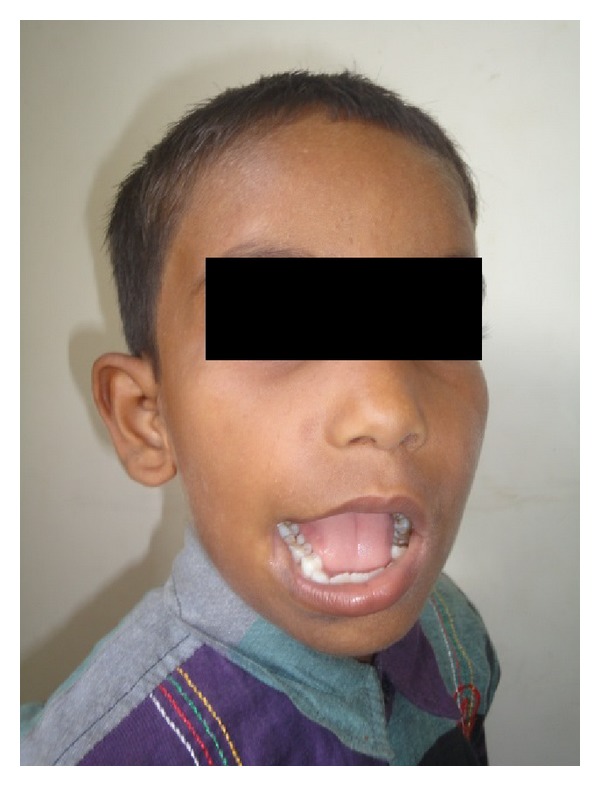
Preoperative photograph with right side ill-formed commissure.

We report a case of 12-year-old boy with a chief complaint of large mouth and drooling of saliva (Figure 1). On evaluation, right commissure of the mouth was ill-formed and preauricular ear tags were observed (Figure 1). The remaining parameters such as occlusion and temporomandibular joint functions are normal. On systemic evaluation, no other skeletal abnormalities were found. Haematological investigations are within normal limits. Patient was posted under general anaesthesia for the correction of ill-formed commissure.

### 2.1. Surgical Technique

**Figure 2:**
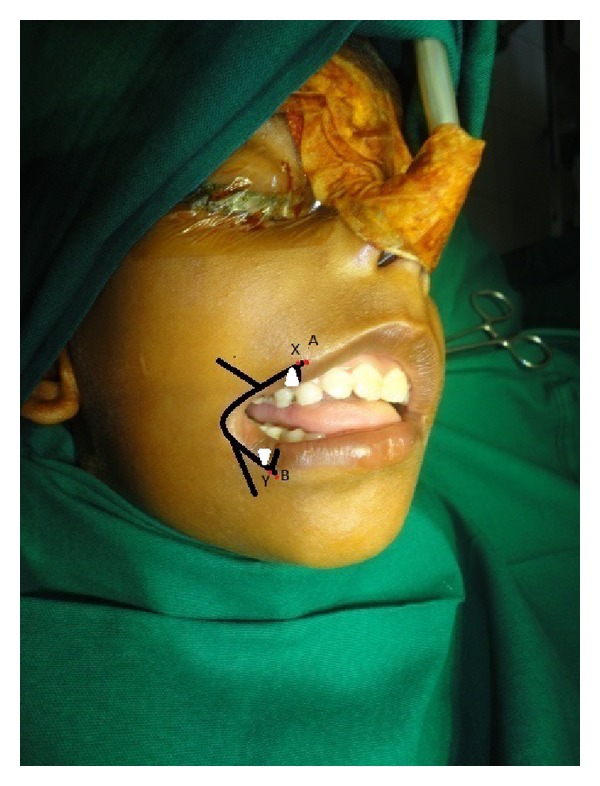
Intraoperative photographs with proposed incision markings showing Z plasty limbs and mucosal flaps (white triangles).

**Figure 3:**
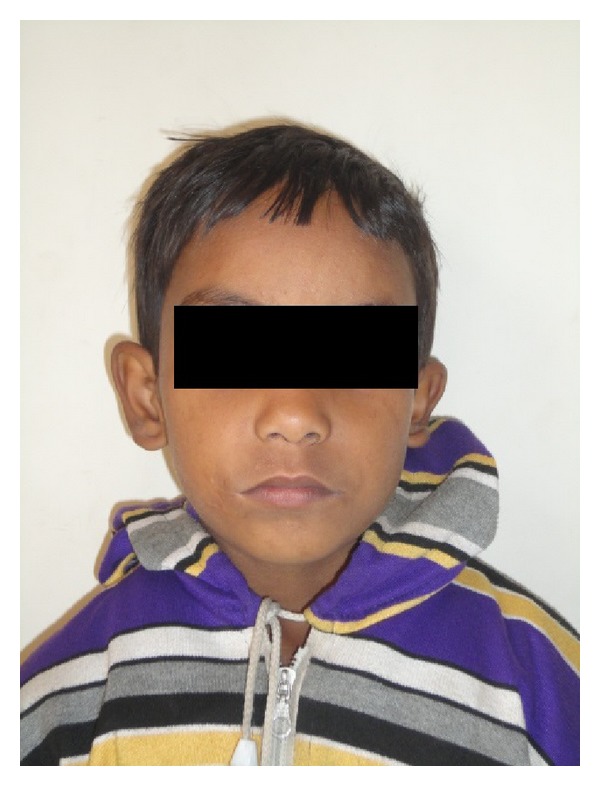
Two-month postsurgery photograph with symmetrical commissure.

**Figure 4:**
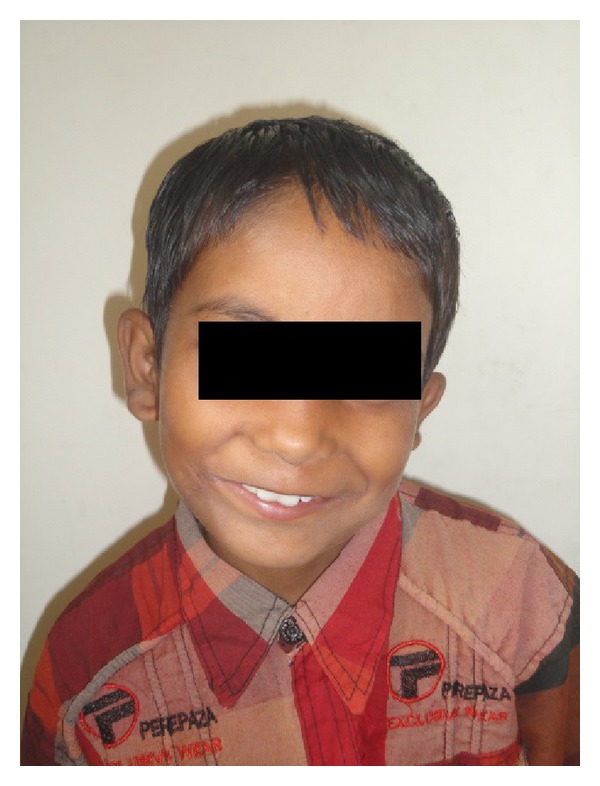
Postsurgery photograph.

Nasal intubation was used for general anaesthesia. Nasal RAE (Ring, Adair, Elwyn) endotracheal tube is used as it will not interfere with evaluation of symmetry of lip. Commissure on the noncleft side is marked. Midpoint of the upper lip is noted at the middle of the peaks of the cupids bow. Lower lip midpoint was determined on a point corresponding to the midline of the upper lip and midline of columella of the nose. On the cleft side, point A was marked on the upper lip and point B was marked on the lower lip at the vermilion cutaneous junction (Figure 2). Two points X and Y were noted 2 mm lateral to the points A and B (Figure 2). Incision on the vermilion mucocutaneous junction extends only up to points X and Y. Mucosal triangular flaps were created by 45° oblique incision lines extending from vermilion mucocutaneous junction of points X and Y. Incision is carried out along the marking. Sterile skin and mucosa are excised. This leaves a V-shaped defect. Orbicularis oris muscle is dissected from labial and mucosal sides. Muscle fibers of upper lip are overlapped on to the lower lip at the commissure. Skin is closed with small Z-plasty. Dog ear which is formed in the closure of the skin is excised. Two-month postsurgical photographs reveal the symmetry of commissures both at rest and at smiling with minimal scar (Figures 3 and 4).

## 3. Discussion

Macrostomia is a rare deformity with variations in its presentation. Defects might range from mild to severe. The extent of clefting in the muscle ranges from mild which is confined to the orbicularis oris to the buccinator or even extends backwards to the masseter muscle. Various techniques were described in the literature for surgical correction. Bütow and Botha [21] gave a classification for the tessier 7 clefts in 2010 as superiorly rotated, middle positioned, inferiorly rotated, or agenic lateral. The severity of these clefts also differs. One should prudently consider these subclassifications to tailor appropriate closure of these clefts blending the scars into natural skin creases. The ideal outcome of surgery is the result of proper placement of scars between different aesthetic subunits of face. The present case is having ill-formed commissure with mild clefting into the orbicularis oris. Reference points from the noncleft side are marked and transposed those into the cleft side to achieve symmetry. We have used triangular mucosal flaps for closure of commissure as it achieves continuous dry red and wet vermilion. Triangular skin flaps for commissure transpose the skin into the corner of mouth and give unnatural appearance. Vermilion square flap method can be used but it is technique sensitive and might give an unsightly scar at vermilion cutaneous junction of the commissure. Overlapping myoplasty is used as it gives the natural overlap of upper lip over the lower lip. Simple straight line closure or improper approximation of muscle bundles might result in fish mouth deformity. The skin is closed with small Z-plasty as the defect is minimal and the limbs of Z-plasty also aligned satisfactorily into the nasolabial and mentolabial folds giving satisfactory healing.

## 4. Conclusion

Various surgical techniques have been proposed for the correction of macrostomia defects. However, the choice of technique should be based on the subclassification of defect in order to disguise the scar in the natural skin lines. Triangular mucosal flaps will result in a natural looking commissure and can avoid transposition of the skin. Overlapping of upper lip muscle fibers on the lower lip muscle fibers will give the natural pouting of lips at rest. Small Z-plasty for skin closure will avoid linear contractures.
